# Vitamin D Deficiency Contributes to the Reduction and Impaired Function of Naïve CD45RA^+^ Regulatory T Cell in Chronic Heart Failure

**DOI:** 10.1155/2015/547697

**Published:** 2015-04-23

**Authors:** Yan-hui Ma, Yun-lan Zhou, Chao-yan Yue, Guang-hui Zhang, Lin Deng, Guo-hua Xie, Wei-ping Xu, Li-song Shen

**Affiliations:** ^1^Department of Laboratory Medicine, Xinhua Hospital, Shanghai Jiao Tong University School of Medicine, Shanghai 200092, China; ^2^Department of Internal Cardiology, Xinhua Hospital, Shanghai Jiao Tong University School of Medicine, Shanghai 200092, China

## Abstract

The effect of vitamin D pertinent to cardiovascular health on the heart itself is considered to shift toward an anti-inflammatory response in chronic heart failure (CHF); however, its underlying mechanism is not completely understood. In this study, we demonstrated that plasma 25(OH)D level, negatively associated with NT-ProBNP, correlated with the decreased Treg in CHF compared to the patients with other cardiovascular diseases and healthy and older donors. Naïve Treg cell (CD4^+^CD45RA^+^Foxp3^lo^T) subset, rather than whole Treg cells, contributes to the reduction of Treg in CHF. 1,25(OH)2D treatment maintained partial expression of CD45RA on CD4^+^T cell after *α*CD3/CD28 monoclonal antibodies activation and ameliorated the impaired CD4^+^CD45RA^+^T cell function from CHF patients through upregulating Foxp3 expression and IL-10 secretion *in vitro*. Low level of vitamin D receptor (VDR) was detected in CD4^+^CD45RA^+^T cell of CHF than control, while 1,25(OH)2D treatment increased the VDR expression to exert its immunosuppression on T cell. The results of this study might provide tangible evidence to our knowledge of the impact of vitamin D supplementation on naïve Tregs, which may offer new means of preventing and treating CHF.

## 1. Introduction

Patients with chronic heart failure (CHF) are characterized by systemic inflammation, as evidenced by raised circulating levels of several inflammatory cytokines such as tumor necrosis factor- (TNF-) *α*, interleukin- (IL-) 1*β*, and IL-6 as well as chemokines, for example, monocyte chemoattractant peptide- (MCP-) 1, IL-8, and macrophage inflammatory protein- (MIP-) 1*α* [1–6], which were associated with high plasma levels of brain natriuretic peptide (BNP) [7]. But the mechanisms of immune activation in CHF remain unknown. Complex interplays between T helper (Th) 1 and Th2 lymphocytes are reported contributing to the pathophysiology of CHF [8]. In addition to Th1 and Th2 cells, there is accumulating evidence for a critical role for Treg and Th17 cell subsets in charge of the immune response and regulation [9]. The proinflammatory cytokine IL-17, which is produced by Th17 cells and other innate immune cells, has been implicated in many inflammatory conditions [10]. Conversely, Treg cells help repress inflammation through modulating T cell responses [11] and can be distinguished from CD4^+^T cell by Foxp3 expression [12, 13]. It has also been proposed that increased Th17 cell levels correlated with decreased Treg cell levels in patients with CHF, suggesting that the imbalance between these two subsets may contribute to the pathogenesis of CHF, which consisted with N-terminal pro-brain natriuretic peptide (NT-ProBNP) [14].

Most peripheral blood Tregs appear as activated phenotype (CD45RA^−^); however, a subset of naïve regulatory T cells (CD45RA^+^) has been detected as well. Previous studies carried out in peripheral blood lymphocytes (PBL) of normal healthy individuals demonstrated three subpopulations (fractions (Fr.) I–III) that expressed Foxp3 and CD45RA protein at different amounts showed distinct phenotype and function [15]. It may explain the reason why human Foxp3^+^Treg cells failed to display homogenous function in previous reports in contrast to murine Foxp3^+^Treg cells [16–19]. The proportion of the three subpopulations differed between normal and pathological conditions [15, 20]. Activated Tregs are highly susceptible to apoptosis and have critically short telomeres, so the peripheral homeostatic mechanisms are very important in charge of Treg diversity and numbers in the maintenance of immune response [21]. Despite the reports of impaired Treg/Th17 balancing, the variation of Treg cell subpopulations in CHF remains to be elucidated.

In recent years, vitamin D was highlighted as an important player in numerous diseases including cardiovascular disorders [22–24]. Vitamin D deficiency may be caused by multiple factors associating with the development and progression of CHF [25–28]. Although vitamin D is a unique nutrition covering a range of pleiotropic effects contributing to the bone and calcium metabolism, its immune modulatory properties are outstanding both* in vivo* and* in vitro* [29–33]. Variation of vitamin D status* in vivo* is associated with the changes of T cell compartment in the human PBL [34]. T cells are known targets for 1,25-dihydroxyvitamin D (1,25(OH)2D), the biological active metabolite of vitamin D, since they express vitamin D receptor (VDR) [35, 36]. Upon T cell activation, the expression of vitamin D receptor is upregulated, suggesting an important functional role for vitamin D in adaptive immunity. Both human* in vitro* and animal models experiments revealed that vitamin D can suppress proinflammatory Th1 and Th17 cytokine responses [37, 38]. How vitamin D works on T cell subsets in CHF is worthy of further exploration.

Herein, we demonstrate the underlying imbalance of Th17 and Treg cell populations in patients with CHF. Reduced serum/plasma concentration of 25(OH)D level, together with elevated NT-ProBNP, was associated with the decreased Treg in CHF. In particular, we assessed variations of Treg subpopulations in CHF and healthy or aged individuals and found that naïve (CD4^+^CD45RA^+^Foxp3^lo^) Treg subset, rather than whole Treg cells, contributes to the reduced Treg in CHF. 1,25(OH)2D supplementary retained the CD45RA expression especially on naïve Treg (CD4^+^CD45RA^+^Foxp3^lo^). It induced Foxp3 expression and IL-17 reduction especially in CD^+^CD45RA^+^T through VDR* in vitro*. Taken together, these results indicated 1,25(OH)2D may be involved in the interaction between Treg and Th17 compartment, skewing T cells into anti-inflammatory and regulatory state in CHF.

## 2. Materials and Methods

### 2.1. Subjects

Between August 2012 and January 2013, in this study, we included 84 patients with CHF, diagnosed by clinical history, physical examination, electrocardiography, echocardiography, chest X-ray, and NT-ProBNP, and 18 patients with acute myocardial infarction (AMI) as other cardiovascular diseases (refer to acute HF) from the Department of Internal Cardiology and Emergency of Xinhua Hospital, Shanghai Jiaotong University School of Medicine. 35 healthy donors (HD) and 15 age- and sex-matched older donors (between 79 and 90 years old) serving as controls were obtained from the medical examination center of Xinhua Hospital. According to New York Heart Association (NYHA) Functional Classification, patients were divided into 3 subgroups: NYHA II, NYHA III, and NYHA IV (for detailed information, refer to Table 1). Exclusion criteria included inflammatory disease, allergic disease, collagen disease, malignant disease, advanced liver disease, renal failure, steroid therapy, and recent myocardial infarction or unstable angina (within 3 months). Written informed consent was obtained from each patient, and the study conformed to the Declaration of Helsinki Principles. The study was approved by the Ethical Committee of Xinhua Hospital affiliated to Shanghai Jiaotong University School of Medicine.

### 2.2. Blood Sampling and Biomarker Measurement

Blood samples used for this study were collected at the time of admission to the Emergency Department or Internal Cardiology Department (<24 h from symptom onset). Plasma 25(OH)D and NT-ProBNP levels, together with hsCRP, LDL, Lp(a), and Hcy, were instantly measured by electrochemiluminescence immunoassay on automated immunoanalyzer (Cobas E601, Roche Diagnostics, Indianapolis, IN) according to the manufacturer's instructions in the clinical chemistry laboratory of Xinhua Hospital. Sera for further measurements were collected and frozen at −80°C until used.

### 2.3. Flow Cytometric Analysis

PBL in the 4 mL heparinized blood were freshly isolated by Ficoll density gradient centrifugation. Lymphocytes were resuspended in PBS supplemented with 2% bovine serum albumin at a concentration of 1 × 10^6^ cells/mL. Cell surface marker analysis was performed using four- or five-color flow cytometric analysis. CD3 eFlour 450 (UCHT1, eBioscience, CA, USA), CD4-PC5 (13B8.2, Beckman Coulter, CA), CD45RA-Cy7 (HI100, Biolegend, San Diego, CA, USA), IL-17A-PE (BL168, Biolegend), CD25-APC (BC96, eBioscience), Foxp3-Ax488 (PCH101, eBioscience), VDR (9A7, abcam), and the secondary antibody were used, together with appropriate isotype controls, to allow identification of positive and negative cell populations. For double staining of IL-17 and Foxp3, PBL were isolated using Ficoll density gradient separation, stimulated with PMA (50 ng/mL) and ionomycin (1 *μ*g/mL) (Sigma-Aldrich), and operated using the Cytofix/Cytoperm kit from BD Biosciences (San Diego, CA, USA) according to the instructions. To analyze the levels of VDR, cells were fixed with 16% formaldehyde (final concentration of approximately 1.5%) for 30 min, permeabilized with 1 mL of ice-cold methanol for 30 min on ice, and stained with rat anti-human VDR monoclonal antibodies and goat anti-human IgG (H+L) secondary antibody DyLight 488. Multiple-color FACS analysis was performed using a BD FACSCanto II flow cytometer (BD). Approximately 1 × 10^4^ to 1 × 10^5^ cells were analyzed; the gating strategy for the identification of T subsets was performed as described previously [39] (Supporting information, Figure S1a in Supplementary Material available online at http://dx.doi.org/10.1155/2015/547697).

### 2.4. Real-Time PCR

Total RNA was isolated with Qiagen reagent. Then, first-strand cDNA was subsequently synthesized using Sensiscript RT Kit (Takara) according to the manufacturer's instructions. Real-time RT-PCR was performed as described previously [40]. The following primers were used to assess gene expression: Foxp3, 5′-CTACGCCACGCTCATCCGCTGG-3′ (forward) and 5′-GTAGGGTTGGAACACCTGCTGGG-3′ (reverse); IL-17A, 5′-AGAGATCCTGGTCCTGCGCA-3′ (forward) and 5′-GTGACACAGGTGCAGCCCAC-3′ (reverse); ROR*γ*t, 5′-ACCACCCCCTGCTGAGAAGGAC-3′ (forward) and 5′-TGCACCCCTCACAGGTGATAACCC-3′ (reverse); VDR, 5′-ATCTGCATCGTCTCCCCAGAT-3′ (forward) and 5′-AGCGGATGTACGTCTGCAGTG-3′ (reverse); GAPDH, 5′-ATTCCACCCATGGCAAATTC-3′ (forward) and 5′-GCATCGCCCCACTTGATT-3′ (reverse).

### 2.5. *In Vitro* Cell Culture

For cell sorting, 10 mL of peripheral blood was collected from either HDs or patients, and CD4^+^T, CD4^+^CD45RA^+^T, CD4^+^CD45RA^−^T, or CD4^+^CD25^−^T cells were obtained from PBL using the human CD4^+^T Cell Isolation Kit II and CD45RA beads (Miltenyi Biotec). The purity of the cells was 90% or greater as determined by reanalysis. Typically, the cells were incubated with 1,25(OH)2D at final concentration 20 nM or DMSO as control under the stimulation with precoated 5 *μ*g/mL *α*CD3, soluble 5 *μ*g/mL *α*CD28, and 200 U/mL rhIL-2 at 1 × 10^5^ per well in 96-well U-bottom plates, and three replicate wells were set up. The cells were cultured in a humidified CO_2_-containing atmosphere at 37°C for 5~7 days in complete RPMI-10 medium supplemented with 100 U/mL penicillin, 100 *μ*g/mL streptomycin, 0.5 mM sodium pyruvate, 0.05 mM nonessential amino acids, 2 mM L-glutamine, and 10 mM HEPES (all from GIBCO). For cell proliferation, purified T cell was stained with CFSE and detected cell division by flow cytometry after 5 days of incubation.

### 2.6. Western Blot and ELISA

Cells were collected after 5 days induced by 1,25(OH)2D or controls for VDR analysis by WB. For cytokine measurements, cell culture supernatants were collected from 24 h to 7 d and diluted for the measurement of cytokines IL-10, IL-17, and IFN-*γ* quantitatively using enzyme-linked immunosorbent assay (ELISA) kits (Multisciences Biotech), according to the manufacturer's instructions. The detection limit of IL-10, IL-17, and IFN-*γ* was 0.7 pg/mL, 15 pg/mL, and 15 pg/mL, respectively.

### 2.7. Statistics

The statistical evaluation was performed with GraphPad Prism (version 5.0, GraphPad Software, CA). Values are shown throughout the paper as mean ± SEM except for the patients and HD age, which is shown as mean ± SD. A Student *t*-test was used to analyze the differences between the groups and one-way ANOVA was initially performed to determine whether an overall statistically significant change existed before using the two-tailed paired or unpaired Student *t*-test for normal distributed data. In the case of significant differences between subgroups, post hoc analyses were based on the Tukey test (normal distributed data) or on the Mann/Whitney *U* test. Pearson's correlation coefficient (normal distributed data) and Spearman's rank correlation coefficient (nonnormal data) were used to assess interrelationships. A *P* value of <0.05 was considered statistically significant.

## 3. Results

### 3.1. Decreased Treg Cells and Elevated Th17 Cells in Patients with Chronic Heart Failure

**Figure 1:**
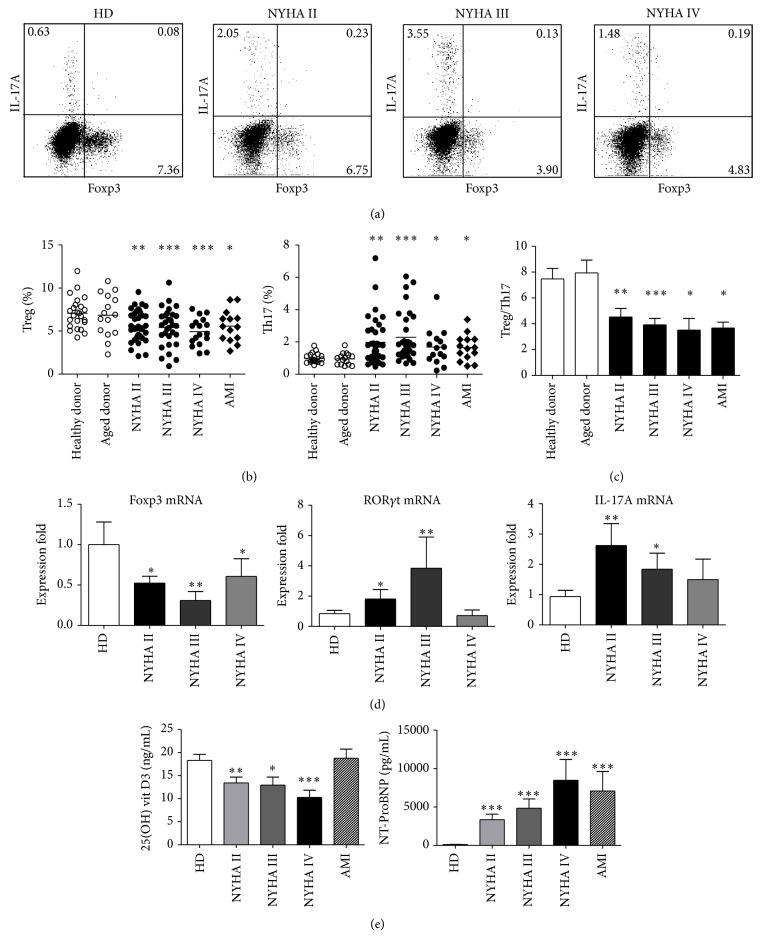
The imbalance of Treg and Th17 cells in patients with CHF. (a) Representative flow cytometric (FCM) dot plots of Foxp3 and IL-17 double staining in CD4^+^T cell. (b) The increased frequencies of Th17 cells and decreased Treg cells were found in CHF patient compared to the healthy donor significantly, while AMI was set as other cardiovascular diseases. (c) The ratio of Treg to Th17 is significantly reduced in CHF. (d) The mRNA expressions of Treg and Th17 related transcription factors and cytokines. (e) Plasma concentrations of 25(OH)D and NT-ProBNP in three CHF groups and controls. Data represent the mean ± SEM; ^*^ *P* < 0.05, ^**^ *P* < 0.01, and ^***^ *P* < 0.001, as compared with HD.

To determine whether Treg and Th17 cells are involved in the development of chronic heart failure, we measured the number of circulating Treg and Th17 cells in PBL by flow cytometry. We defined the phenotype of Treg cells as CD4^+^CD25^+^Foxp3^+^. As shown in Figures 1(a) and 1(b), the proportion of Tregs of HDs ranged from 3.1% to 11.9%, compared with 1.0% to 10.5% in the patients with CHF. The frequencies of Treg cells were significantly reduced in patients with CHF (NYHA II 5.63 ± 0.30%, NYHA III 5.43 ± 0.40%, and NHYA IV 4.95 ± 0.38%) compared to healthy donors (7.08 ± 0.37%) and elder donors (6.85 ± 0.64%) (*P* < 0.05). The proportion of Th17 cells in the total CD4^+^T cells in HDs ranged from 0.6% to 1.8%, while the percentages in patients with CHF were from 0.7% to 7.2%. The frequencies of Th17 cells were markedly higher in patients associated with clinical stage (NYHA II 1.98 ± 0.24%, NYHA III 2.29 ± 0.28%, and NHYA IV 1.68 ± 0.29%) compared with the healthy donors (0.99 ± 0.07%) and elder donors (0.95 ± 0.09%) (*P* < 0.05, Figures 1(a) and 1(b)). Overall, the data showed a decrease in Treg cells and an increase in Th17 cells in CHF. An imbalance was observed in patients with CHF and AMI (acute HF) (*P* < 0.01), which was characterized by lower frequencies of Tregs and higher frequencies of Th17 cells in CD4^+^T cells, as shown by the ratio of Treg/Th17 cells (Figure 1(c)). There were equivalent numbers of frequencies of Treg and Th17 cells observed in HD controls and elder donors. Further, there was no significant difference between percentages of Treg and Th17 cells in the CHF patients and AMI as AHF controls (*P* > 0.05). Similar to cytometry results, Foxp3 and TGF-*β* mRNA expressions of PBL were reduced in three NYHA groups, while ROR*γ*t and IL-17A mRNA levels were significantly higher in CHF patients compared with HDs (Figure 1(d)). Multicolor flow cytometry showed CD4^+^CD45RA^−^T cell producing IL-17 mostly (Supporting information, Figure S1b).

### 3.2. Association between Vitamin D Status, NT-ProBNP, and the Frequencies of Treg and Th17

**Figure 2:**
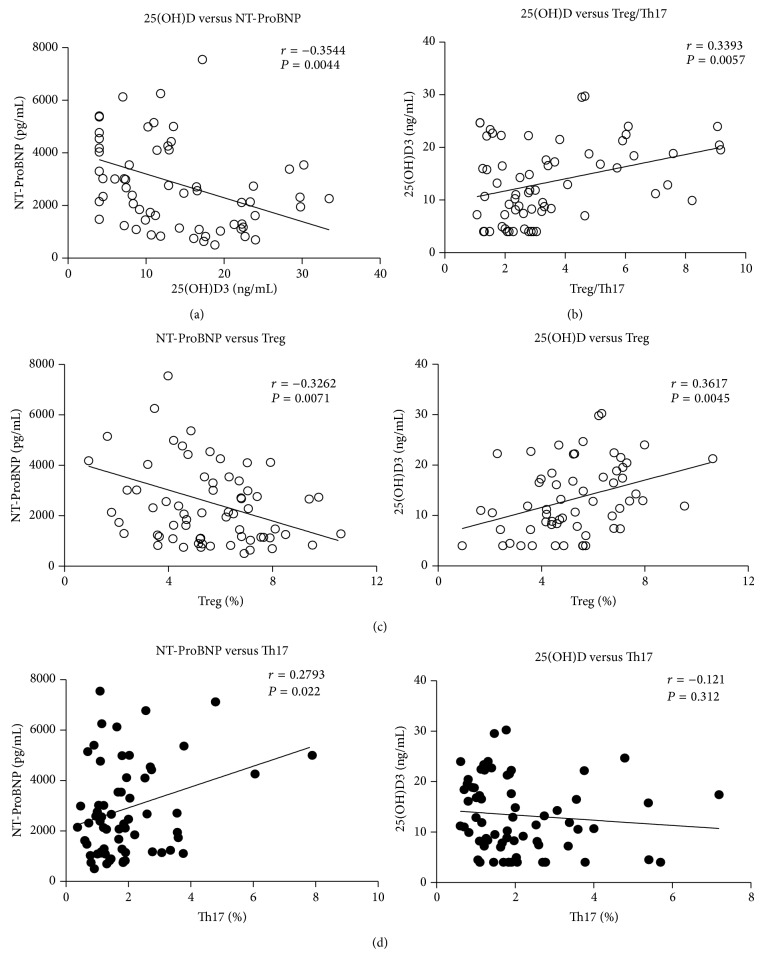
The correlation between Treg or Th17 and 25(OH)D or NT-ProBNP in chronic HF. (a) 25(OH)D was correlated with Treg/Th17 and NT-ProBNP. (b) The significant decreased frequency of Treg in patients with chronic HF positively correlated with 25(OH)D and negatively correlated with NT-ProBNP. (c) The frequency of Th17 positively correlated with NT-ProBNP but not correlated with 25(OH)D.

Accumulating evidence suggests that vitamin D deficiency is associated with cardiovascular disease (CVD) and excess mortality [41]. To assess whether 1,25(OH)2D status correlated with Treg and/or Th17, we first assessed concentration of plasma 25(OH)D and plasma NT-ProBNP from CHF patients classified into three NYHA groups, AMI (refer to acute HF) controls, and aged donors. NT-ProBNP is a sensitive marker for cardiac dysfunction, elevated NT-ProBNP levels indicated patients with ventricular dysfunction, and levels correlate directly with the severity of heart failure [42]. Compared to AMI and aged donor, levels of 25(OH)D were lower in all groups of CHF patients (Figure 1(e)). Moreover, lower 25(OH)D and higher NT-ProBNP were consistent with increased clinical severity. NT-ProBNP, which is associated with ventricular dysfunction, was inversely correlated with 25(OH)D (*r* = −0.3544, *P* = 0.0044) (Figure 2(a)). Given our findings of reduced ratio of Treg/Th17 cell populations in patients with CHF, it was correlated with plasma level of 25(OH)D as well (*r* = 0.3393, *P* = 0.0057) (Figure 2(b)). We also found that the frequency of Treg was negatively correlated with NT-ProBNP (*r* = −0.3262, *P* = 0.0071) and positively correlated with 25(OH)D (*r* = 0.3617, *P* = 0.0045) (Figure 2(c)). Meanwhile, the frequency of Th17 was positively correlated with NT-ProBNP (*r* = 0.2793, *P* = 0.022) but not significantly correlated with 25(OH)D (*r* = −0.121, *P* = 0.312) (Figure 2(d)).

### 3.3. Variations of Human Naïve Treg and Activated Treg Cell Populations under CHF Disease Conditions

**Figure 3:**
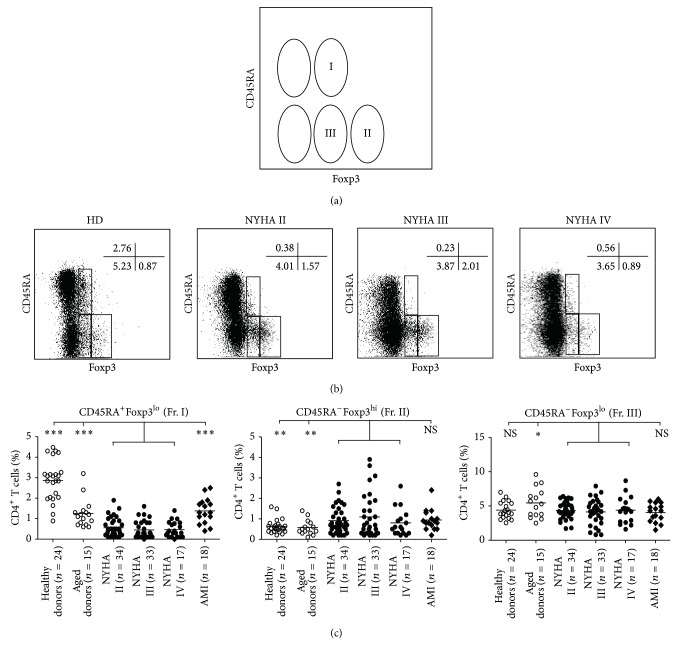
Variations of Foxp3^+^ cell subpopulations under physiological and CHF conditions. (a) Flow cytometry of PBL gated on CD4^+^T cells isolated from PBL. A schematic of T cell subpopulations separated based on Foxp3 and CD45RA. (b) Flow cytometry of PBL gated on CD4^+^T cells isolated from a healthy adult, an old donor, and three patients from each clinical stage. Percentage of each quadrant in each staining combination is shown. (c) Percentages of each Foxp3^+^ subset among CD4^+^T cells in indicated numbers of patients with CHF NYHA II/III/IV, AMI, aged donors (between 79 and 90 years old), and healthy donors (between 18 and 60 years old). Black horizontal bars represent mean percentage. Data represent the mean ± SEM; ^*^ *P* < 0.05, ^**^ *P* < 0.01, and ^***^ *P* < 0.001.

The combination of Foxp3 and CD45RA staining of CD4^+^T cells in PBL of healthy individuals revealed three subsets, that is, Foxp3^lo^CD45RA^+^ cells (Fr. I), Foxp3^hi^CD45RA^−^ cells (Fr. II), and Foxp3^lo^CD45RA^−^ cells (Fr. III) notably (Figure 3(a)). The proportion of naïve Treg cells (Fr. I) among CD4^+^T cells was decreased in CHF (0.50% ± 0.41%, *n* = 84 versus 2.86% ± 0.99% in healthy donors, *n* = 24; *P* < 0.0001) whereas that of activated Treg cells (Fr. II) was increased (0.84% ± 1.21% versus 0.48% ± 0.77%; *P* = 0.0011; Figures 3(b) and 3(c)). We also applied the new definition of Foxp3^+^T cell subsets to the analysis of aging condition. In older people, naïve Treg was lower than normal individuals but higher than patients with CHF (1.25% ± 0.69%, *n* = 15 versus 0.50% ± 0.41%, *n* = 84; *P* < 0.0001), and CD45RA^−^Foxp3^lo^ non-Treg cell fraction (Fr. III) increased compared to CHF (5.44% ± 1.25% versus 4.27% ± 1.62%; *P* = 0.0166) and healthy donors. There was no difference in activated Treg between healthy and older donors. According to the analysis of T cell population, we assumed CD45RA as a marker for the functional T cells on 1,25(OH)2D activity.

### 3.4. 1,25(OH)2D Maintains CD45RA Expression on CD4^+^CD45RA^+^T Cells after *α*CD3/CD28 Stimulation

**Figure 4:**
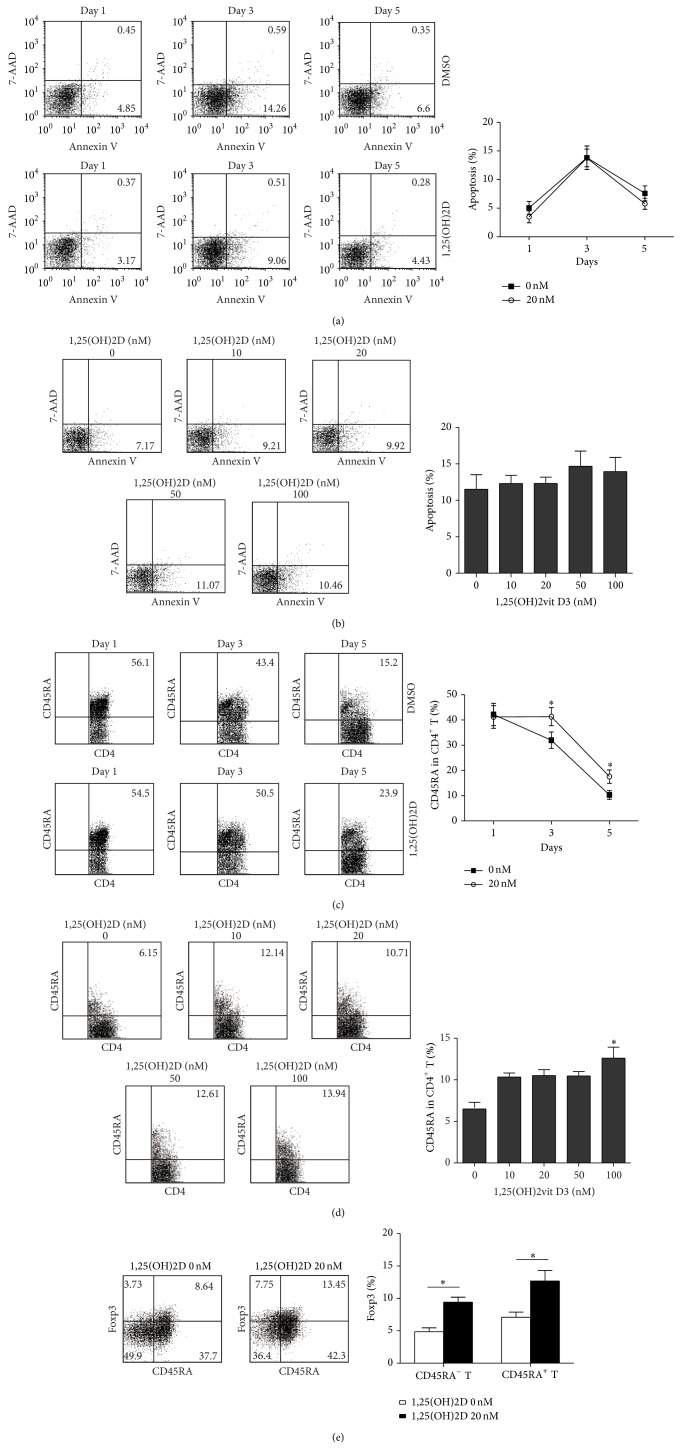
The impact of 1,25(OH)2D on T cell. (a) PBMCs purified from HD were activated by *α*CD3/CD28 with 20 nM 1,25(OH)2D or DMSO. Cells were collected on days 1, 3, and 5 and analyzed apoptosis in CD4^+^T cell by FACS. (b) PBMCs were cultured as described with a series of concentrations of 1,25(OH)2D from 10 nM to 100 nM and observed apoptosis in CD4^+^T cell on day 3. (c) CD4^+^T cell purified from HD were activated by *α*CD3/CD28 monoclonal antibodies with 20 nM 1,25(OH)2D or DMSO. Cells were collected on days 1, 3, and 5 and analyzed CD45RA expression by FACS. (d) CD4^+^T cell from CHF was cultured as described with a series of concentrations of 1,25(OH)2D from 10 nM to 100 nM and observed CD45RA expression. (e) CD45RA and Foxp3 expressions in CD4^+^T cell with or without vitamin d supplement on day 3. Data are representative of three independent experiments.

To assess the effects of high dose vitamin D3 supplementation on T cell, we treated the cells with a series of concentrations of 1,25(OH)2D and detected the cell apoptosis. T cells from HD cultured with *α*CD3/CD28 and 20 nM 1,25(OH)2D for 5 days showed similar apoptosis compared with DMSO (Figure 4(a)). High dose 1,25(OH)2D (50 or 100 nM) increased T cells apoptosis slightly on day 3 (Figure 4(b)). CD45RA expression on T cells from HD cultured with 20 nM 1,25(OH)2D was significantly higher than DMSO control after *α*CD3/*α*CD28 activation on day 3 and day 5 (*P* < 0.05, Figure 4(c)). 1,25(OH)2D maintained the CD45RA, to some extent, in a dose dependent manner. High dose 1,25(OH)2D had a significant role on CD45RA expression (*P* < 0.05, Figure 4(d)).

### 3.5. 1,25(OH)2D Promotes Foxp3 Expression While It Inhibits IL-17A in CD4^+^CD45RA^+^T Cells in CHF Patients

**Figure 5:**
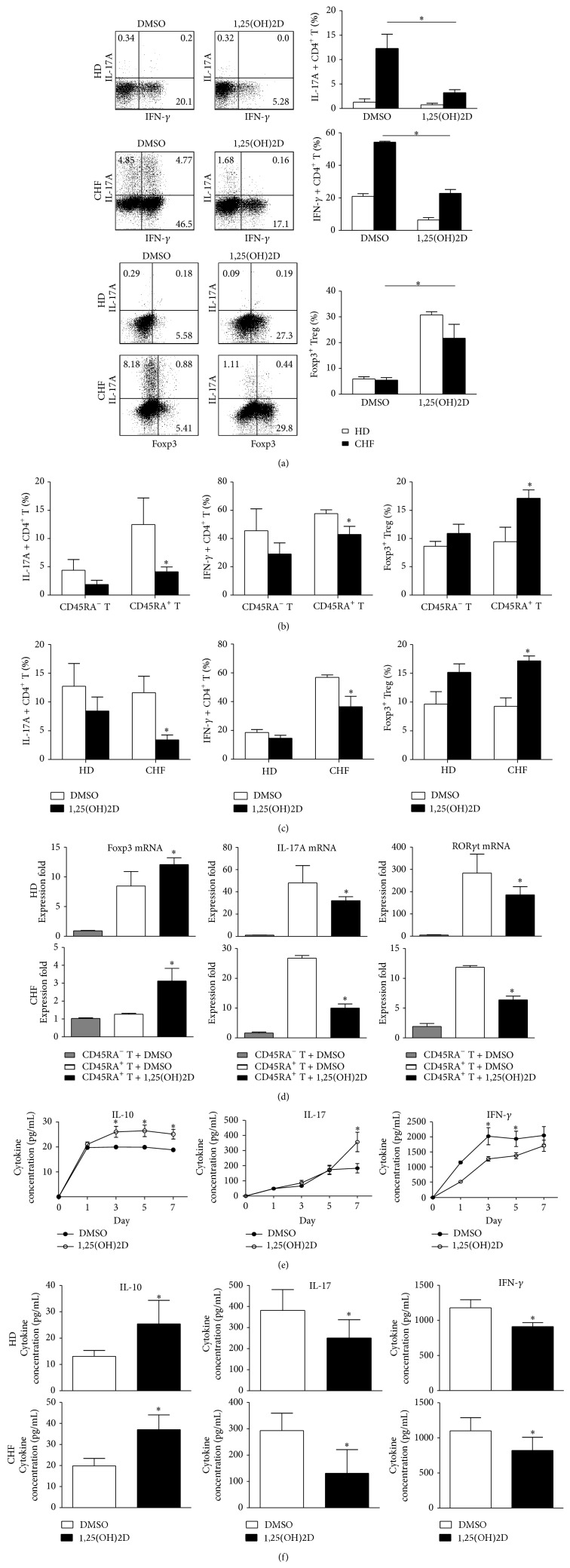
1,25(OH)2D elevated Foxp3 expression while it inhibits IL-17A in CD4^+^CD45RA^+^T cells. (a) Representative flow cytometric dot plots and diagrams of IFN-*γ* and IL-17 or Foxp3 and IL-17 double staining in CD4^+^T cell after 5 days of culture with 1,25(OH)2D or DMSO. (b) CD4^+^CD45RA^+^T and CD4^+^CD45RA^−^T cells were purified from HD. IL-17, IFN-*γ*, or Foxp3 expressions were detected by FACS after 7 days of culture. (c) CD4^+^CD45RA^+^T cells were purified from HD and CHF. IL-17, IFN-*γ*, or Foxp3 expressions were detected by FACS after 7 days of culture. (d) Foxp3, IL-17, and ROR*γ*t mRNA expressions in CD4^+^CD45RA^+^T cells after 7 days of culture, CD45RA^+^CD4^−^T cells as negative control. (e) HD CD4^+^CD45RA^+^T cells culture supernatant was collected on days 0, 1, 3, 5, and 7 and detected IL-10, IL-17, and IFN-*γ* by ELISA. (f) CD4^+^CD45RA^+^T cells were purified from HD and CHF. IL-10, IL-17, and IFN-*γ* were detected by ELISA on day 7. Data are representative of five independent experiments. Results shown are the mean ± SEM; ^*^ *P* < 0.05.

Previous results showed dramatically decreased naïve Treg cell in CHF. To confirm the function of 1,25(OH)2D on T cells, CD4^+^T cells were first purified from HD and CHF and were cultured with 20 nM 1,25(OH)2D or DMSO in the presence of *α*-CD3, *α*-CD28, and rhIL-2. Results showed that the percentage of CD45RA^+^Foxp3^+^ naïve Treg cells was higher after 1,25(OH)2D treatment for 3 days in PBMCs from HD (*P* < 0.05, Figure 4(e)). CHF CD4^+^T cell with 1,25(OH)2D treatment expressed less IL-17 and IFN-*γ* (Figure 5(a) top) and more Foxp3 than HD (Figure 5(a) bottom). We further found that CD4^+^CD45RA^+^T cells were the major population reacting to the 1,25(OH)2D in HD, as 1,25(OH)2D significantly inhibited IL-17 and promoted Foxp3 expression on CD4^+^CD45RA^+^T cells than CD4^+^CD45RA^−^T cells (Figure 5(b)). The same response and mRNA expression pattern of CD4^+^CD45RA^+^T cells in CHF compared with HD. It showed lower IL-17 and higher Foxp3 expression (Figure 5(c)) and lower IL-17 and ROR*γ*t mRNA and higher Foxp3 mRNA expression in CD4^+^CD45RA^+^T cells (Figure 5(d)). Elevated IL-10 and decreased IL-17 and IFN-*γ* were found in the supernatants of HD CD4^+^CD45RA^+^T cells cultured with 1,25(OH)2D in 7 days in series (Figure 5(e)). Cytokine profile of CD4^+^CD45RA^+^T cells culture supernatants in CHF was the same as in HD (Figure 5(f)). No obvious changes in anti-inflammatory cytokine TGF-*β* were observed (data not shown). Above all, results showed that 1,25(OH)2D induced CD4^+^CD45RA^+^T cells convert to Foxp3^+^iTreg, produce more IL-10, and inhibit IL-17 secreting.

### 3.6. 1,25(OH)2D Induced CD4^+^CD45RA^+^T Cells Prohibited CD4^+^CD25^−^T Effector Cells via Upregulated VDR

**Figure 6:**
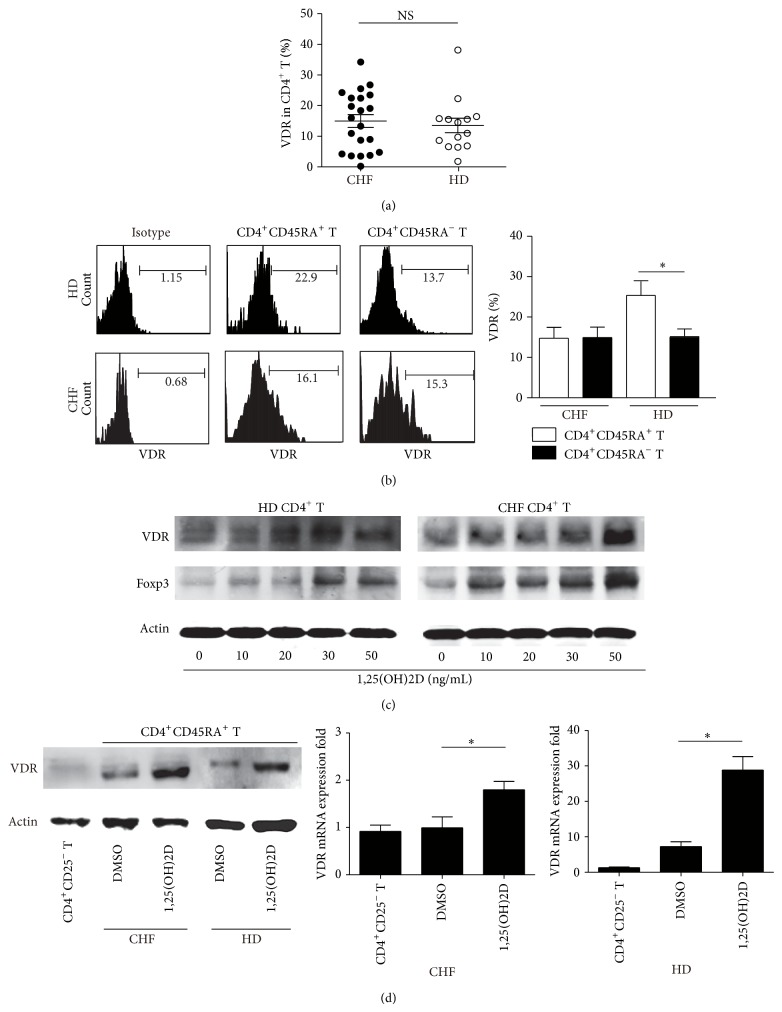
1,25(OH)2D induced CD4^+^CD45RA^+^T cells prohibited CD4^+^CD25^−^ T effector cells via upregulated VDR. VDR expression in CD4^+^T (a) or in CD4^+^CD45RA^+^T and CD4^+^CD45RA^−^T cells (b) was measured by FACS PBL from PBL of HD and CHF. (c) CD4^+^T cells were isolated by MACS and then incubated with a series of concentrations of 1,25(OH)2D for 5 days. VDR and Foxp3 expression in induced cells was measured by WB. (d) CD4^+^CD45RA^+^T cells were isolated by MACS and incubated with 1,25(OH)2D or DMSO for 5 days. VDR expression was measured by WB and real-time PCR.

**Figure 7:**
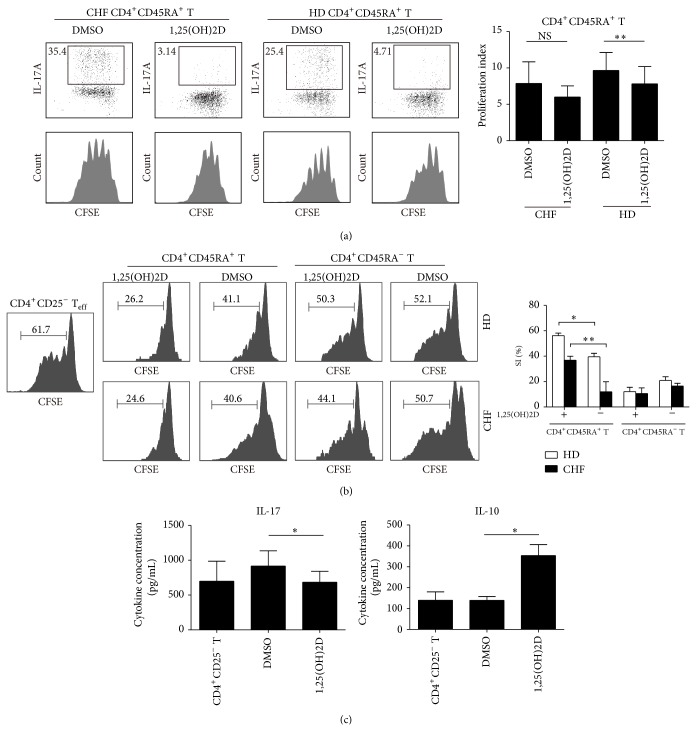
1,25(OH)2D ameliorates the impaired immunomodulation of CD4^+^CD45RA^+^T cell. (a) CD4^+^CD45RA^+^T cells were purified from HD and CHF, labeled with CFSE subsequently, and incubated with 1,25(OH)2D or DMSO for 5 days. Representative flow cytometric dot plots and histograms of CFSE and IL-17A double staining. (b) Suppression of responder T cell proliferation was preceded using transwells. 1,25(OH)2D induced CD4^+^CD45RA^+^T or CD4^+^CD45RA^−^T cells were placed into top chamber while  CD4^+^CD25^−^T cells were added into bottom chamber. After 5 days, cells were stained for CD4 and proliferation of CFSE-labeled responder T cells was analyzed on a flow cytometer. (c) The level of IL-10 and IL-17 in culture supernatant on day 5 was detected by ELISA. Data are representative of five independent experiments. Results shown are the mean ± SEM; ^*^ *P* < 0.05.

1,25(OH)2D exerts its biological function depending on the VDR [43], and we found there was no difference of VDR expression on CD4^+^T cell between HD and CHF (Figure 6(a)). Further analysis showed that CD4^+^CD45RA^+^T cells expressed more VDR than CD4^+^CD45RA^−^T cells in HD, while it was not found in CHF (Figure 6(b)). CD4^+^T cells were isolated and incubated with 1,25(OH)2D or DMSO for 5 days; VDR and Foxp3 expression was upregulated in a dose dependent manner (Figure 6(c)). Purified CD4^+^CD45RA^+^T cells expressed higher level of VDR when cultured with 1,25(OH)2D than those with DMSO both in CHF and HD (Figure 6(d)). CD4^+^CD45RA^+^T cells from HD and CHF were labeled with CFSE and incubated with 1,25(OH)2D or DMSO for 5 days and we analyzed their proliferation index. We found that CD4^+^CD45RA^+^T cells treated with 1,25(OH)2D expanded at slightly slower pace (for CHF *P* = 0.098; for HD *P* = 0.002) (Figure 7(a)). IL-17 expression dropped sharply in CD4^+^T cells, especially in CD4^+^CD45RA^+^T cells when cocultured with 1,25(OH)2D. The transwell coculture system was used to show the suppression of 1,25(OH)2D induced CD4^+^CD45RA^+^T cells on CD4^+^CD25^−^T effector cells (T_eff_). It is noteworthy that CD4^+^CD45RA^+^T cells apart from HD exhibited a certain degree of suppression, but it would be reinforced in the presence of 1,25(OH)2D (*P* = 0.016) (Figure 7(b) upper). CD4^+^CD45RA^−^T cells induced by 1,25(OH)2D or DMSO did not show any suppression on T_eff_ (Figure 7(b) upper). CD4^+^CD45RA^+^T cells apart from CHF had little suppressive function, while in the presence of 1,25(OH)2D these cells could restore their inhibition on T_eff_ (*P* = 0.012) (Figure 7(b) lower). Also, increased IL-10 and decreased IL-17 were detected in the 1,25(OH)2D culture supernatant compared with DMSO (*P* < 0.05) (Figure 7(c)).

## 4. Discussion

Vitamin D status has been linked to chronic heart failure (CHF) either in large clinical trial studies or in experimental* in vitro* and animal studies. However, the potential role of vitamin D in immunological deregulation in cardiac dysfunction is not well understood and remains to be elucidated. This is the first study to investigate the correlation between vitamin D status and the composition of T cell compartment and subpopulations of Foxp3^+^Treg* in vivo* in CHF patients.

CHF is considered to be a complex multistep disorder in which a number of physiologic systems participate in its pathogenesis [44]. Recent studies have provided strong lines of evidence implicating that the activation of the immune system and the prevalence of inflammation contribute to the progression of CHF. Increased plasma/serum inflammatory cytokines and chemokines are significantly correlated with deterioration of cardiac function (i.e., New York Heart Association classification) and performance (e.g., left ventricular ejection fraction (LVEF)) [2–5]. Moreover, these inflammatory mediators may also provide important prognostic information to CHF [45]. Although the mechanisms for the inflammation are unknown, a growing body of evidence suggests that Treg and Th17 cells may play a role in the inflammation. The initial aim of this study was to determine whether an imbalance between Th17 and Treg cell populations is characteristic of CHF, as previously suggested [14]. We confirmed that increased Th17 and decreased Treg cell population frequencies correlated with the development of clinical stages. The ratio of Treg/Th17 was lower in patients with advanced CHF. Besides, we also showed reduced Foxp3 and TGF-*β* expression and elevated ROR*γ*t and IL-17A expression accordingly. These results suggested that Treg/Th17 imbalance may participate in the development and progression of CHF.

Previous evidence demonstrated impaired Th1/Th2 balance in patients with CHF despite various etiologies [8, 46]. With further understanding of immune activation and modulation, the Treg/Th17 cell balance seems to be more notable and convertible due to immunoregulatory therapy. As reported, human CD4^+^Foxp3^+^T cells can be separated into three phenotypically and functionally distinct subpopulations: CD45RA^+^Foxp3^lo^ naïve Treg cells (Fr. I), CD45RA^−^Foxp3^hi^ activated Treg cells (Fr. II), and CD45RA^−^Foxp3^lo^ nonsuppressive T cells (Fr. III) [15]. In CHF patients, we found the proportion of Fr. I T cells (naïve Treg) was decreased as compared to normal individuals, resulting in relatively elevated Fr. II, as the absolute count of Fr. II was not significantly higher than that of HD (data not shown). These variations were in accord with patients' clinical stages (NYHA). Taking into account the influence of age, we also analyzed three Foxp3^+^T cell subsets in aged healthy subjects. Although the frequency of Fr. I was lower than that of normal individuals, aged individuals still had higher frequency of Fr. I and lower frequency of Fr. II than that of CHF. The variations in human Treg cell populations under CHF conditions may be highly informative in assessing the severity of disease. Foxp3^hi^ activated Treg appears to be terminally differentiated Treg cells; it may be converted from naïve Treg or CD4^+^ Foxp3^−^T cell [21] and rapidly die* in vitro* [15]. Nevertheless, naïve Treg could be potentially proliferative upon stimulation. Thus, the sharp drop of naïve Treg in CHF may explain their subdued immunomodulation. Future research needs to determine underlying mechanism of how vitamin D gives rise to naïve Treg cells persistence* in vivo* constantly in adults.

Studies have linked vitamin D deficiency to the development and progression of CHF [24–28]. Vitamin D undergoes two hydroxylations to form the active metabolite, 1,25-dihydroxyvitamin D, termed calcitriol, and acts as a modulator of calcium homeostasis and the immune response [47–49]. Vitamin D treatment in CHF decreased serum concentrations of TNF-*α*, an inflammatory cytokine; in contrast, increased concentrations of IL-10, an anti-inflammatory cytokine, suggested that vitamin D has protective effects on the heart itself [26, 27]. However, clinical trial on vitamin D treatment showed no effect on left ventricular function compared to placebo treatment in men with CHF [50]. Thus, the effect of vitamin D pertinent to cardiovascular health is the shift toward an anti-inflammatory response [51].

As reported, high prevalence of vitamin D insufficiency (<30 ng/mL) was found in 84% males and 89% females in China. The overall median (interquartile range) serum concentration of 25(OH)D was 20.9 (16.9–25.0) ng/mL [52], which consisted with plasma 25(OH)D concentration in this study. Technically, it does not verify whether this criterion applied to Asians to assess vitamin D levels is practical. We found levels of plasma 25(OH)D were lower in all groups of CHF patients compared to AMI, aged, and healthy donors. In our study, 25(OH)D was inversely correlated with NT-ProBNP, which was associated with ventricular dysfunction and consisted with increased clinical severity. Additionally, we showed declined ratio of Treg/Th17 cell populations in patients with CHF; 25(OH)D was positively correlated with the frequency of Treg, whereas it was not significantly correlated with the frequency of Th17. Further results showed that CD4^+^CD45RA^+^T cell was the population that better responded to 1,25(OH)2D treatment, which partially restored its suppressive function against inflammation in CHF.

Naïve Treg cells (CD4^+^CD45RA^+^Foxp3^+^T) manifested equivalent suppressive activity to activated Tregs (CD4^+^CD45RA^−^Foxp3^+^T)* in vitro*. Targeting naïve Tregs in adults may offer new means of preventing and treating immune-related disease [53]. Herein, we employed CD4^+^CD45RA^+^T instead of CD4^+^CD25^+^CD45RA^+^T cell, which was considered equal to CD4^+^CD45RA^+^Foxp3^lo^ T cell, as a marker to illustrate the effect of vitamin D on T cells [15, 39]. Besides the fact that not enough CD4^+^CD45RA^+^Foxp3^lo^ cell was available for harvesting from CHF, the other major reason was that we considered CD45RA as a more practical marker to investigate the role of vitamin D on T cell subsets as inducible Foxp3 expression after 1,25(OH)2D treatment. As reported, naïve T (mostly CD45RA^+^T) cell could express Foxp3 in the presence of 1,25(OH)D; herein, we also found that CD4^+^CD45RA^+^T cell expressed more Foxp3 and less IL-17 after 1,25(OH)D treatment. Vitamin D supplementation increased naïve CD4^+^T cells preferentially and induced a significant increase of regulatory T cells [54]. Results from CD4^+^T cell experiment showed CD45RA^−^Foxp3^+^T cell elevated as well. Besides the role of 1,25(OH)2D on Foxp3 expression, the consequent drop of CD45RA expression, after activation, in T cell with 1,25(OH)2D was less considerable on the detecting time point, leading to the percentage of Foxp3^+^Treg in CD45RA^−^T probably being relatively higher. The *α*CD3/CD28 stimulation is artificial and gives a very strong response, so CD45RA^−^Foxp3^+^T cell will be the dominant population at last. VD-iTreg would be more appropriate to decribe the Foxp3^+^Treg induced by 1,25(OH)2D [55].

Through vitamin D receptor (VDR), 1,25(OH)2D inhibits proliferation of T effector cells, reduces the production of IL-2 and IFN-*γ* and cytotoxicity [56, 57], and decreases the synthesis of IL-6, IL-12, IL-17, and IL-23 [58–60]. Hence, it seems likely that 1,25(OH)2D suppresses the generation of Th1 and Th17 cells and probably induces the development of Foxp3^+^Treg cells. Due to vitamin D supplements being not practicing in clinical treatment of CHF or other cardiovascular diseases as an adjuvant therapy in China, it was difficult to assess vitamin D status and T cell subpopulations* in vivo* for following up or achieve conclusion whether the change in the density of CD4^+^CD45RA^+^Foxp3^lo^T cells in CHF patients is the cause or consequence of low 25(OH)D concentration. VDR is preferentially ligated with retinoid X receptor (RXR) to form heterodimers to bind DR3-type sequences [61] or bind genomic DNA first and then forms complexes with corepressor proteins and histone deacetylases to mediate gene transcription [62]. The presence of VD response elements (VDREs) in the intronic conserved noncoding sequence region of the human* foxp3* gene (+1714 to +2554) and the enhancement of the* foxp3 *promoter activity by such VDREs in response to 1,25(OH)2VD were reported [55]. More VDR ChIP-seq data, especially in primary CD4^+^T cell [63], provide interesting and helpful information. Our findings addressed reduced VDR expression in CD4^+^CD45RA^+^T cells in CHF; however, the role of vitamin D on CD45RA expression through VDR or other mechanisms needs further study.

Taken together, this work demonstrates that decreased vitamin D is correlated with decreased Treg and increased Th17 cells in CHF. There are also variations of Foxp3^+^Treg subpopulations with remarkably declined CD4^+^CD45RA^+^Foxp3^lo^ naïve Treg cells and arisen CD4^+^CD45RA^−^Foxp3^hi^ activated Treg cells in CHF. More importantly, 1,25(OH)2D has the ability to ameliorate the impaired immunomodulation of CD4^+^CD45RA^+^T cell on T_eff_ in CHF through VDR. Our findings provide tangible evidence to vitamin D supplementation, as a nonpharmacologic strategy, which may help prevent the development and progression of CHF through immunoregulation.

## Supplementary Material

Figure S1: Flow cytometry analysis of T cells.

## Figures and Tables

**Table 1:** Characteristics of chronic heart failure patients and healthy donors.

	HD	Chronic HF			Acute myocardial infarction
		NYHA II	NYHA III	NYHA IV	
*n*	35	34	33	17	18
Gender (M/F)	17/18	20/14	28/7	8/9	13/5
Age, mean ± SD years	45.1 ± 11.6	77.7 ± 11.1	79.2 ± 9.4	78.1 ± 6.4	72.4 ± 14.8
HR (bmp)	NA	83 ± 23	85 ± 18	85 ± 16	81 ± 16
LVEF (%)	NA	61.6 ± 9.6	59.4 ± 11.3	51.4 ± 11.9	52.5 ± 17.4
LVEDD (d/mm)	NA	52.0 ± 6.7	54.5 ± 9.3	55.6 ± 8.3	51.1 ± 4.1
HF risk factors, *n* (%)					
Hyperlipemia	NA	7 (20.6)	6 (18.2)	2 (11.8)	2 (11.1)
Diabetes mellitus	NA	16 (47.1)	14 (42.4)	5 (29.4)	7 (38.9)
Cigarette smoking					
Former smoker	NA	7 (20.6)	6 (18.2)	3 (17.6)	3 (16.7)
Current smoker	NA	4 (11.8)	9 (27.3)	1 (5.9)	1 (5.6)
Disease etiology, n (%)					
Coronary heart disease	NA	20 (58.8)	22 (66.7)	7 (41.2)	11 (61.1)
Hypertension	NA	22 (64.7)	28 (84.8)	7 (41.2)	10 (55.6)
Cardiomyopathy	NA	2 (5.9)	1 (3.0)	2 (11.8)	0 (0)
Biochemical cardiac markers					
25(OH)D (ng/mL)	17.7 ± 6.9	13.4 ± 7.6^**^	12.9 ± 9.3^*^	10.3 ± 6.5^***^	19.8 ± 7.9
NT-ProBNP (pg/mL)	NA	2466 ± 2327	3994 ± 4886	3690 ± 3025	3591 ± 3691
Hcy (mmol/L)	NA	10.67 ± 0.96	17.57 ± 3.70	22.83 ± 6.57	10.68 ± 1.52
hsCRP (ng/mL)	0.39 ± 0.07	7.25 ± 0.86^***^	9.13 ± 0.73^***^	8.32 ± 0.93^***^	10.31 ± 0.96^***^
LDL (mmol/L)	2.61 ± 0.11	1.92 ± 0.12^**^	1.89 ± 0.12^**^	1.77 ± 0.16^**^	1.96 ± 0.27^**^
Lp(a) (mg/dL)	6.34 ± 1.35	6.32 ± 0.92	9.95 ± 1.63	8.25 ± 2.18	11.40 ± 2.26
Medications, *n* (%)					
ACEI/ARB	NA	6 (17.6)	12 (36.4)	2 (11.8)	3 (16.7)
*β*-blockers	NA	8 (23.5)	9 (27.3)	3 (17.6)	5 (27.8)
Digitalis	NA	4 (11.8)	6 (18.2)	7 (41.2)	2 (11.1)
Diuretics	NA	9 (26.5)	9 (27.3)	8 (47.1)	4 (22.2)
Statins	NA	6 (17.6)	6 (18.2)	2 (11.8)	6 (33.3)
Ca^2+^-blockers	NA	6 (17.6)	5 (15.2)	1 (5.9)	0 (0)

Except where indicated otherwise, values are the number (%). NA = not applicable. Data represent the mean ± SD. ^*^ *P* < 0.05, ^**^ *P* < 0.01, and ^***^ *P* < 0.001 compared to HD.
