# Supplementary material for: Vitamin D Deficiency Contributes to the Reduction and Impaired Function of Naïve CD45RA+ Regulatory T Cell in Chronic Heart Failure
Source: J Immunol Res. 2015 Apr 23;2015:547697. doi: 10.1155/2015/547697 (PMC4423006; doi:10.1155/2015/547697)
Supplement: Supplementary file 1 — Figure S1: Flow cytometry analysis of T cells. [file 547697.f1.pdf]

Supporting information

Fig.S1 Flow cytometry analysis of T cells. (a) Routine analysis of FCM data on CD45RA, Foxp3, CD25 and IL-17A on T cells labelled with anti-CD3-eFlour 450 and anti-CD4-PC5. Gate 1 presented lymphocytes the in light scatter plot. Gate 2 presented CD4+T cells. CD45RA^{+}Foxp3^{lo} naive Treg cells (Fr.I), CD45RA^{-}Foxp3^{hi} activated Treg cells (Fr.II), and CD45RA^{-}Foxp3^{lo} nonsuppressive T cells (Fr.III) analyzed in Gate 3,4 and 5, respectively. (b) Representative flow cytometric dot plots of IL-17 and CD45RA double staining in CD4 T cell.

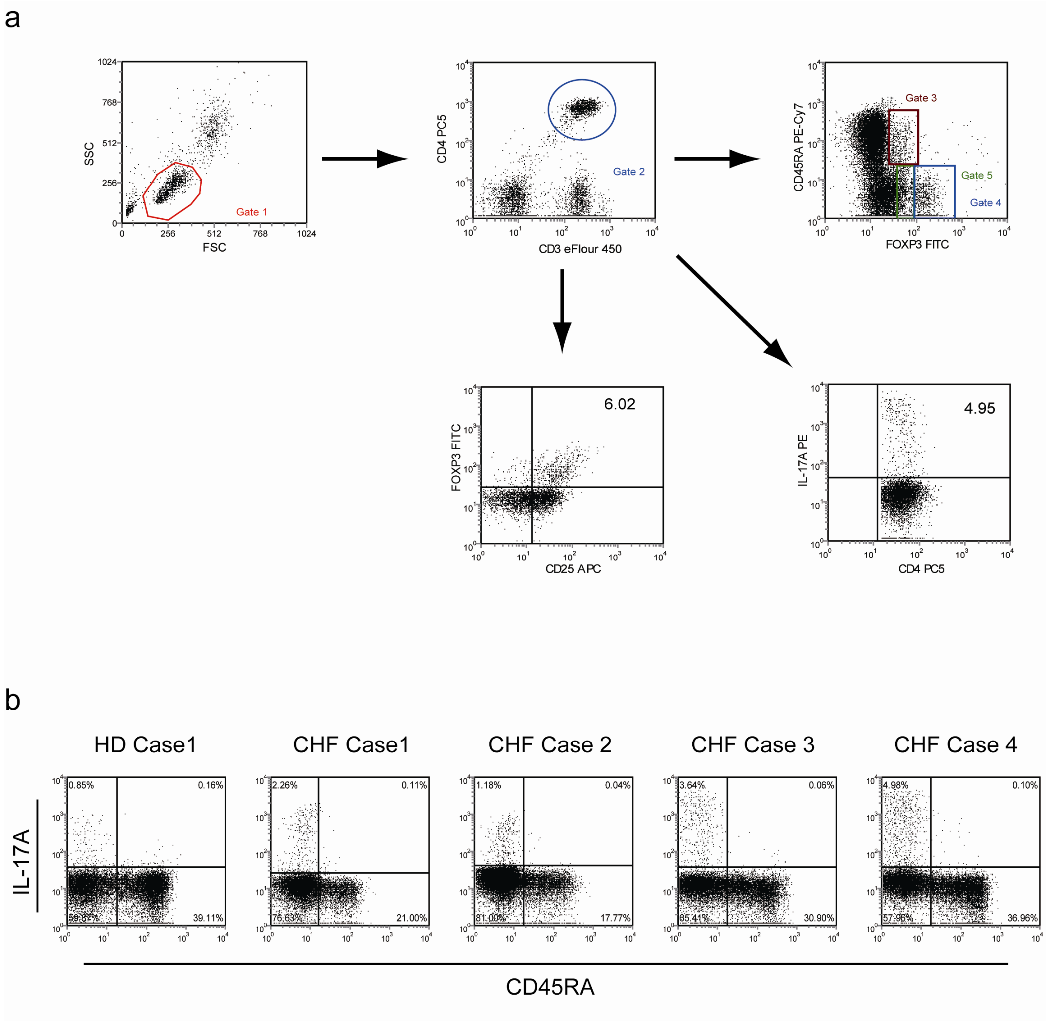

Fig.S1
